# Extending Regulatory Biokinetic Lead Models towards Food Safety: Evaluation of Consumer Baby Food Contribution to Infant Blood Lead Levels and Variability

**DOI:** 10.3390/foods12142732

**Published:** 2023-07-18

**Authors:** Skyler A. Price, Mark A. Maddaloni, Brent L. Finley, Stephanie A. Thornton, Ken M. Unice

**Affiliations:** 1 Stantec ChemRisk, Brooklyn, New York, NY 11201-1217, USA; skyler.price@stantec.com (S.A.P.);; 2 Stantec ChemRisk, Pittsburgh, PA 15222-4801, USA

**Keywords:** PBTK model, lead (Pb), infants, blood lead levels (BLLs), probabilistic assessment, baby food, dietary lead

## Abstract

The U.S. Food and Drug Administration released proposed lead (Pb) action levels for foods intended for babies and young children in January 2023 based on the agency’s interim reference value of 2.2 µg/day for dietary Pb. Since the 1980s, biokinetic models have estimated blood lead levels (BLLs) associated with environmental contamination, but their use in food safety assessment has been limited. We compared three recent biokinetic models (IEUBK Model, ICRP Model Version 5, and AALM) to develop insights on contributors to variability in potential exposures to Pb in consumer baby food products. While modest variation was observed for babies, the predictions trended to convergence for children aged 3 and older, approaching the U.S. FDA dietary conversion factor of 0.16 µg Pb/dL blood per µg Pb intake/day. We applied the IEUBK model in a probabilistic exposure assessment framework characterizing the distribution of Pb in soil, dust, water, and food intake in the United States. Soil and dust were the primary contributors to variance in infant BLLs, while food and water contributed <15% combined. Thus, reductions in upper-bound soil and dust concentrations will be necessary before achieving appreciable reductions in the frequency of BLLs greater than the BLRV of 3.5 µg/dL.

## 1. Introduction

Several studies have reported the presence of heavy metals, including lead (Pb), in baby food products in the United States (U.S.), including a 2021 U.S. House of Representatives Subcommittee on Economic and Consumer Policy report that detailed high levels of inorganic arsenic, lead, cadmium, and mercury in baby food products [1,2,3,4]. The subcommittee concluded that baby food products are a source of heavy metal exposure for infants and young children and pose a threat to children’s neurological development [4]. Lead, a metal that has long been the subject of regulations, was found in baby food products or ingredients for baby food products produced by all the responding companies. Approximately 19% of Nurture (HappyBABY) products contained more than 10 ppb Pb, and Beech-Nut, Hain (Earth’s Best Organic), and Gerber products all used ingredients with detectable Pb content [4]. In children, Pb is able to cross the blood–brain barrier by substituting for calcium ions, allowing the metal to enter and accumulate in the child’s developing central nervous system [5]. Additionally, infants and young children experience an estimated 5 to 10 times greater gastrointestinal absorption of Pb compared to adults [6,7]. Elevated blood lead levels (BLLs) in children have been associated with various neurological and behavioral effects, including motor performance, language development, aggression, and depression [8].

More recently, in January of 2023, the U.S. Food and Drug Administration (FDA) released draft guidance on action levels for Pb in baby food products to minimize the likelihood that a consumer will be exposed to Pb levels in food exceeding the U.S. FDA’s Interim Reference Level (IRL) [9] (U.S. Food and Drug Administration, 2023). If finalized, these action levels will represent the official stance of the U.S. FDA on Pb in baby food products. The basis of the proposed Pb action levels is the U.S. FDA’s IRL for infants and young children, which was updated to 2.2 µg/day in 2022 [10]. This IRL was derived from the U.S. Center for Disease Control’s (CDC) Blood Lead Reference Value (BLRV) of 3.5 µg/dL, using a conversion factor of 0.16 µg/dL per µg Pb/day and a 10-fold safety factor [10]. The dietary conversion factor was calculated by the U.S. Environmental Protection Agency (EPA) in 1986 using data from Ryu et al. (1983) [11] where infant Pb exposure was estimated based on known exposure to Pb from infant formula, and subsequently compared to BLL estimates generated by a Pb biokinetic model [12,13]. The U.S. FDA 2014–2016 Total Diet Survey indicates that children of ages 1 to 3 ingest 1.7 (mean) to 2.6 µg/day (90th percentile) of dietary lead, which corresponds to incremental BLL increase of 0.27 to 0.42 µg/dL, respectively, using a dietary conversion of 0.16 µg/dL per µg Pb/day [14].

Biokinetic models of Pb have been extensively used to support contaminated site and building exposure and risk assessments by estimating and describing the physiological distribution of Pb throughout the human body. These models estimate blood Pb concentrations based on a series of exposure parameters and assumptions that inform the model’s inputs. Since the 1980s, multi-compartmental models have been developed to more accurately estimate Pb uptake and distribution throughout the whole body [15].

Several biokinetic models are currently available, although only a subset of these models is capable of estimating BLLs in infants and children. The Integrated Exposure Uptake Biokinetic Model (IEUBK) is a multicompartment model that estimates BLLs in children (0–7 years) by independently modelling exposures from air, food, water, soil, and dust [16]. The absorption from these multiple compartments is then modelled independently and combined as a single input into the blood plasma reservoir [16]. Like the IEUBK Model, the International Commission for Radiation Protection (ICRP) model and All-Ages Lead Model (AALM) are also multicompartmental but possess the advantage of modeling Pb kinetics over the entire human lifetime. The ICRP and AALM models are also capable of modeling transient changes in BLLs resulting from short-term or intermittent exposures, whereas the IEUBK algorithm is based on pseudo steady-state kinetics which places minimum requirements on exposure frequency (once weekly) and duration (3 months) to run the model.

To date, few studies have compared the outputs and methodologies of available Pb biokinetic models, particularly for young children and infants. Pounds and Leggett (1998) [17] provide a direct comparison between the contemporaneous ICRP and IEUBK models, using the IEUBK default Pb intake and uptake values as the inputs for both models to estimate BLLs in children from birth to seven years of age. The authors found that the ICRP model produced BLL estimates that were approximately twice the IEUBK model estimates [17]. Lakind (1998) [18] compared the IEUBK Lead99 model, the O’Flaherty model, and the Carlisle and Wade model using intermittent exposure of children to Pb-contaminated soil at an active firing range as a case study. This analysis found that the O’Flaherty model was the most suited for assessing episodic exposures to Pb, and that the IEUBK Lead99D and the Carlisle and Wade model likely overestimated childhood BLLs when using these models to simulate episodic exposures [18].

To our knowledge, no peer-reviewed study has directly compared the IEUBK, ICRP, and AALM biokinetic models. Furthermore, no study has compared the output and sensitivity of these models with respect to estimating BLLs in infants. The aim of this current study is to use the proposed U.S. FDA action levels as a case study to compare the IEUBK, ICRP, and AALM biokinetic models, present a framework for the application of biokinetic models to estimate BLLs in infants consuming baby food products, and compare the model outputs to the U.S. FDA’s estimates in the proposed draft guidance for Pb in baby food.

## 2. Materials and Methods

The overall aims of this assessment are first to transparently compare and evaluate model predictions for dietary intake of Pb in baby foods and secondarily to assess the relative importance of soil, dust, water, and food intake to a recent nationally observed distribution of BLLs.

### 2.1. Dietary Conversion Factor

The dietary conversion factor has been used by the FDA to relate the unit intake of dietary Pb to the incremental increase in BLL in young children assuming roughly linear responses at dietary intake levels (µg Pb/dL blood increase in BLL per 1 µg Pb intake per day) [10,12]. This factor was compared to that predicted using recent versions of the IEUBK and ICRP models, as well as the most recently updated AALM model. These models were selected to represent variation in model structure, calibration processes, and assumed kinetics (Table 1). The most recent versions of the U.S. EPA IEUBK model Version 1.1 Build 11 (2010) and Version 2.0 Build 1.66 (2017) were obtained from the U.S. EPA Superfund website (Lead at Superfund Sites: Software and Users’ Manuals|US EPA). The ICRP biokinetic model version 4 from Syracuse Research Corporation with the dimension of the intake estimates increased to 3000 (R5CHELAT.3000) was provided by Roy and Edwards (2022) [19]. The U.S. EPA extensions of ICRP version 5 and subsequent preparation of AALM Version 2.0 (also referred to as ICRP version 8 in model generated files) were obtained from the U.S. EPA Lead (https://www.epa.gov/lead/approach-estimating-exposures-and-incremental-health-effects-lead-due-renovation-repair-and, accessed on 13 March 2023) and Science Inventory (All-Ages Lead Model (AALM), Version 2.0 (External Review Draft, 2019)|Science Inventory|US EPA) websites, respectively. The conversion factor was assessed using each of the models for the early childhood life stages of 0.5 to <1, 1 to <2, 2 to <3, and 3 to <7 years old for intakes of either 1 µg/day or 5 µg/day (Table 2), which were selected to bound the currently recommended FDA interim reference level for Pb of 2.2 µg/day corresponding to a BLL of 0.35 µg/dL [10]. The upper bound of 5 µg/day is more than 4-fold less than the 22 µg/day intake corresponding to the CDC BLRV of 3.5 µg/dL when the FDA dietary conversion factor is applied (i.e., 22 µg/day × 0.16 µg/dL per µg Pb/day = 3.5 µg/dL).

### 2.2. Biokinetic Model versus Conversion Factor Estimated BLLs

The most recent versions of the models that included calibration data representative of children (IUEBK Version 2.0, ICRP version 5, and AALM Version 2 were used to estimate BLLs for 5 µg/day Pb dietary intake for comparison to BLLs estimated using the FDA dietary conversion factor). Average BLLs were predicted for the same age groupings previously adopted of 0.5 to <1, 1 to <2, 2 to <3, and 3 to <7 years, as well as a continuous time-concentration profile from age 0 to 7.

### 2.3. Probabilistic Assessment

Recently compiled U.S. national distributions of Pb in soil, dust, water, and food presented in the peer-reviewed literature provide the opportunity to assess the contributions of these sources to observed population variance in observed BLLs in children through the use of probabilistic, or Monte Carlo analyses, such as those described by the U.S. EPA Risk Assessment Forum [20]. Probabilistic analyses in risk assessment are intended to provide range and likelihood of hazard, exposure, or risk, rather than the more limited information available from a single point estimate. In the case of Pb intakes, variability in the human population exists in intake as well as in the biokinetic response between individuals with similar intakes. The focus of the present analysis is on the distribution of central tendency BLLs associated with variability in media concentrations (soil, water, and dust) and food intake, as previous assessments, including the development and evaluation of IEUBK, have examined variability between individuals and measurement error characterized by a geometric standard deviation of 1.6 [16,21].

#### 2.3.1. Soil, Dust, Water, and Food Distributions

The distributions of soil, dust, and water concentration as well as food Pb intake in the United States were assumed to be lognormally distributed based on data recently compiled (Table 2). Soil and dust concentrations were based on 2005–2006 American Healthy Homes Survey (AHHS; n-n = 942) as summarized in Zartarian et al. (2017) [22] and HUD (2011) [23]. Food Pb intakes were based on the Food and Drug Administration (FDA)’s Total Diet Study (TDS) data collected between 2007–2013 and FDA’s mapping food items from the TDS to the dietary intakes recorded in the National Health and Nutrition Examination Survey (NHANES) [22,24]. Water concentrations were based on a summarization of the AHHS II of U.S. homes conducted between 2018 and 2019 (n = 678) [25]. Soil and dust measurements were also collected in AHHS II and reported in the CDC final report but have not yet been published in the peer reviewed literature [26]. Soil and dust distributions were segregated by (1) pre-1950 or (2) 1950 and later construction with approximately 17% of homes considered to be pre-1950 construction (Table 2). Airborne concentrations of lead were set to the IEUBK default values because prior studies have shown air to contribute 1% or less to intakes [27].

#### 2.3.2. Soil, Dust, Water, and Food Correlations

Within homes, weak to moderate correlations exist between dust, soil, and water concentrations associated with underlying determinants such as the year of home construction, construction characteristics and practices, as well as setting such as urban or rural or near and far from high traffic areas (Table 2). Rank correlation coefficients of 0.48 for soil–dust pairs and 0.2 for dust–water and soil–water pairs were assigned [22]. Similarly, within an individual, repeated measures or characterizations of food intake are likely to be moderately correlated. A rank correlation of 0.7 reflecting moderate year–year correlation in food intake was assumed based on moderate correlations observed in repeated measures of BLLs in children [28].

#### 2.3.3. IUEBK Monte Carlo Assessment

The predicted national distribution of central tendency BLLs (i.e., accounting for variability in media concentrations, but no interhuman biokinetic variability) was based on 10,000 iterations drawn from the input distributions and correlations between distributions presented in Table 2. Input distributions were prepared using Argo Version 4.1.3, 64-bit (Booz Allen Hamilton, Inc., McLean, VA, USA). Following the preparation of 10,000 unique probabilistic intake scenarios in Argo Version 4.1.3, the distribution of geometric mean BLLs corresponding to the probabilistically assigned soil, dust, and water Pb concentrations as well as food Pb intake were estimated in IEUBK Version 2 batch mode.

Three scenarios were evaluated, including a (1) baseline scenario, (2) a 30% reduction in the distribution of dietary Pb intake scenario, and (3) a 50% reduction in soil Pb concentration distribution scenario. The 30% reduction in dietary Pb intake scenario is intended to evaluate the potential decrease in early childhood Pb ingestion following implementation of the U.S. The FDA (2023) [9] proposed an action level of 10 ppb for fruits and vegetables, as well as 20 ppb for root vegetables and dry infant cereals. At the 90th exposure percentile, the agency has estimated that with these action levels, dietary Pb intake would decrease from a baseline of 2.02 µg/day to 1.5 µg/day (~26% reduction for sum of fruits, root vegetables, and dry infant cereal). The 50% reduction in soil Pb concentration is intended to evaluate the observed decrease in mean soil Pb concentration in homes with children under the age of 6 from 172 to 83 ppm (~50% reduction) in the 2005–2006 AHHS and 2018–2019 AHHS II surveys, respectively. 

Correlations between BLLs and the exposure concentrations or food Pb intake were calculated, and the overall percent contribution of the exposure concentrations or food intake was assessed using the first-order variance-based estimation algorithm of Plischke (2012) [29], which uses the discrete cosine transformation (DCT) originally applied in digital image processing. Percent contributions to variance were normalized to 100%. The first-order effect describes the contribution to variance independently attributable to each predictor. Total parameter sensitivity inclusive of interactions can also be estimated [30,31,32], but were not evaluated in this study because the first-order (main) effects appear to explain most of the variance in model predictions. 

The probabilistic results were interpreted based on descriptive statistical summaries of the 10,000 iterations. Visualizations focused on intakes segregated by predicted BLLs less or greater than the CDC BLRV of 3.5 µg/dL were also prepared. The predicted distribution of BLLs was compared to BLLs reported in the National Health and Nutrition Examination Survey.

**Table 1:** Overview of aim, development, and attributes of historical and current multimedia exposure models for the prediction of BLLs in children.

Consideration	IEUBK v1.1/v2	ICRP v4	ICRP v5	AALM
Aims	Prediction of the likely BLL distribution for children of ages 6 to 84 months from environmental exposures.	Multicompartmental occupational tissue/organ predictions of occupational Pb biokinetics to age 60.	Multicompartmental general child and adult tissue/organ predictions of Pb biokinetics to age 60.	Predictions of lifetime Pb concentrations in blood, other body tissues, and excreta for ages 0 up to 90.
Modules	Intake, uptake, biokinetics	Intake, uptake, biokinetics	Intake, uptake, biokinetics	Intake, uptake, biokinetics
Defined Pathways	Water, Food, Soil, Dust, Air	Injection, Inhalation, Oral	Water, Food, Soil, Dust, Air	Water, Food, Soil, Dust, Air
BLL prediction	Geometric mean and %-ile	Central tendency	Central tendency	Central tendency
Bone lead prediction	No	Yes (mineral weight basis)	Yes (mineral weight basis)	Yes (mineral weight basis)
Calibrated w/child data	Yes	No	Yes	Yes
Calibration data	Autopsy study of children; two field studies.	Radiolabel studies of humans; animal data.	2007–2008 National Health and Nutrition Examination Survey; occupational blood and bone lead studies conducted in 1994, 1999, and 2008.	Occupational studies, human child and adult volunteer studies with known dose, and post-mortem soft tissue data.
Integration time step	Pseudo-steady state (month)	User-defined (e.g., 0.1 day)	User-defined (e.g., 0.1 day)	User-defined (e.g., 0.1 day)
Preceding modeling framework	Infant and juvenile baboon calibrated model [33]. Version 0.99d considered human data in children [34]	ICRP model for bone-seeking radionuclides [17,35].	Update of ICRP v4.	Update of ICRP v5.
Key changes from preceding framework	Version 1.1 reflected minor updates to dietary lead intake, maternal blood lead, and bone weight [36]. Version 2.0 updates dietary lead intake, drinking water consumption, maternal blood concentration, inhalation rates, and soil/dust ingestion rates [37].	Version 1 added chelation; Version 2 added output for bone Pb wet weight converted to dry weight as µg of Pb per g of bone mineral; Version 3 converted output time from days to years, and Version 4 added output options.	Version 5 added scaling of tissue mass by age, updated bone lead transfer rates, and calibration data considering both children and adults.	AALM added growth parameters; revised gastrointestinal absorption factors, and kinetic parameter refinement.
Default Pb media concentration or intakes	Yes; soil and dust defaults characterized as “starting values” modifiable by site specific data.	No	No	Described in Technical support document [38]
Gastrointestinal absorption factor (%)	Food and water: 50%; Soil and dust: 30%	Varies from 45% to 15% decreasing with age	Varies from 45% to 15% decreasing with age	Varies from 39% to 12% decreasing with age
Relative bioavailability	Included in absorption factor	None by default	None by default	Soil dust RBA = 60%
Model reference	U.S. EPA, 2007; U.S. EPA, 2021a	Leggett, 1993; Pounds and Leggett, 1998	U.S. EPA 2014	USEPA 2019b

**Table 2:** Exposure assumptions considered in the (a) model dietary unit intake response comparison, (b) IEUBK probabilistic assessment.

Parameter	Unit ^a^	Distribution	Parameter 1 ^b^	Parameter 2 ^b^	Probabilistic Median ^a^ [5th–95th %-ile] ^c^	Reference
(a) Unit BLL concentration (incremental BLL per unit increase in oral Pb intake						
Dietary Pb intake	µg/day	Point	1 or 5	--	--	Hypothetical
Other Pb intake	µg/day	Point	0	--	--	Dietary only
(b) Distributions of predicted BLLs using the IEUBK model for national scenario						
Food Pb intake						
…age 0 to 0.5	µg/day	Lognormal	AM = 0.70	SD = 0.98	0.41 [0.07–2.3]	[22]
…age 1 to <2	µg/day	Lognormal	AM = 2.6	SD = 1.8	2.1 [0.73–6.0]	[22]
…age 2 to <3	µg/day	Lognormal	AM = 3.4	SD = 2.0	3.0 [1.2–7.3]	[22]
…age 3 to <4	µg/day	Lognormal	AM = 3.5	SD = 2.1	3.1 [1.3–7.4]	[22]
…age 4 to <5	µg/day	Lognormal	AM = 3.6	SD = 2.2	3.1 [1.2–7.7]	[22]
…age 5 to <6	µg/day	Lognormal	AM = 3.9	SD = 2.2	3.4 [1.4–8.0]	[22]
…age 6 to <7	µg/day	Lognormal	AM = 3.8	SD = 2.0	3.4 [1.5–7.6]	[22]
Correlation age i to j	--	Spearmans’s r	0.7	--	--	[39]
Soil and Dust						
…Homes built < 1950	fraction	Bernoulli	*p* = 0.169	--	--	[40]
…Dust built < 1950	ppm	Lognormal	AM = 196	SD = 212	--	[22] ^d^
…Dust built ≥ 1950	ppm	Lognormal	AM = 75	SD = 52	--	[22] ^d^
…Dust combined	ppm	Calculated	--	--	67 [23–255]	
…Soil built < 1950	ppm	Lognormal	AM = 505	SD = 1062	--	[22] ^d^
…Soil built ≥ 1950	ppm	Lognormal	AM = 42	SD = 59	--	[22] ^d^
…Soil combined	ppm	Calculated	--	--	30 [4.7–455]	[22]
Correlation soil–dust	--	Spearmans’s r	0.48	--	--	[22]
Drinking water						
Drinking water	ppb	Lognormal	AM = 1.0	SD = 2.4	0.39 [0.04–3.7]	[25]
Correl. soil–water	--	Spearmans’s r	0.2	--	--	[22]
Correl. dust–water	--	Spearmans’s r	0.2	--	--	[22]

^a^ denotes not applicable. ^b^ AM = Arithmetic mean; SD = Standard deviation, *p* = occurrence probability. -- denotes not applicable. ^c^ Column represents the median, 5th and 95th percentile estimated in the Monte Carlo simulation based on the assumed parameters. ^d^ Based on fitted log mean and log standard deviation reported by authors.

## 3. Results

### 3.1. Dietary Conversion Factor

The models’ predicted BLL units were equal to or exceeded the U.S. FDA dietary con-version factor of 0.16 (µg/dL per µg/day) up to age 3 (Table 3). The IEUBK, ICRP, and AALM model predictions generally differed from each other by less than 10 to 15%, except for the ICRP v4 model, which was not specifically calibrated for children (Table 1). In general, the U.S. FDA dietary conversion factor appears to be more similar for children from 3 to 7 years old than for children younger than 3. Thus, for a given intake of dietary lead, BLLs in very young children might increase on average somewhat more than predicted by the factor considered in the U.S. FDA’s derivation of the interim reference level. 

**Table 3:** Unit BLL dietary conversion factor (µg/dL blood) per (µg/day intake) for IEUBK, ICRP, and AALM models, compared to the EPA conversion factor used to develop the U.S. FDA proposed action levels for Pb in baby food products.

Dietary Intake (µg/day)	Age	Dietary Intake to BLL Conversion Factor (µg/dL per µg/day)						FDA Conversion Factor ^a^
		IEUBK v1.1	IEUBK v2	ICRP v4	ICRP v5	AALM		
Sex ->		M/F	M/F	M/F	M/F	M	F	M/F
1	0.5 to <1	0.28	0.28	0.45	0.26	0.29	0.31	0.16
	1 to <2	0.23	0.22	0.33	0.19	0.24	0.25	0.16
	2 to <3	0.19	0.19	0.28	0.16	0.21	0.22	0.16
	3 to 7	0.16	0.16	0.25	0.14	0.16	0.17	0.16
5	0.5 to <1	0.27	0.27	0.39	0.24	0.27	0.28	0.16
	1 to <2	0.22	0.22	0.30	0.18	0.23	0.24	0.16
	2 to <3	0.19	0.19	0.26	0.15	0.21	0.22	0.16
	3 to <7	0.16	0.16	0.24	0.14	0.16	0.16	0.16

^a^ Originally calculated by U.S. EPA using data on infants’ BLLs and known exposure to Pb from cartons or cans of formula (see Flannery et al., 2020 [12]).

### 3.2. Predicted BLL Model Comparison

**Figure 1:**
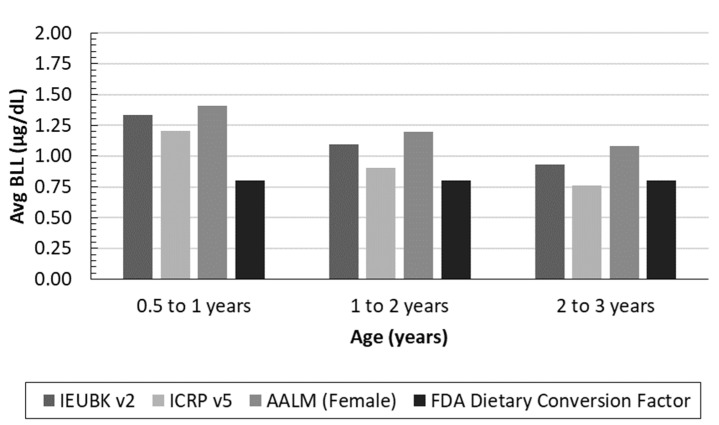
Average BLLs estimated for children aged 0.5–1, 1–2, and 2–3 years consuming 5 µg/day dietary intake for model comparison.

**Figure 2:**
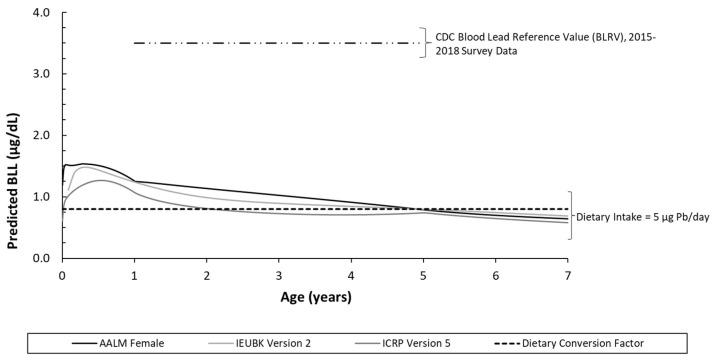
Time blood lead biokinetic model concentration profile corresponding to 5 µg/day total dietary intake as compared to the FDA dietary conversion factor for children ages 0 to 7.

The three models specifically calibrated for children, or IEUBK Version 2.0, ICRP Version 5, and AALM Version 2.0 (female), predicted similar central tendency or mean BLLs for infants and young children hypothetically ingesting 5 µg/day dietary Pb (Figure 1). The IEUBK v2 and AALM predicted BLLs were in close agreement (e.g., approximately 1.25 µg/dL for a 0.5 to 1 year old child), whereas the U.S. FDA dietary conversion factor prediction was lower than any of the three models calibrated for children (e.g., 0.8 µg/dL irrespective of child’s age). The predicted BLL profile from birth to age seven (Figure 2) illustrates the agreement of the models across several early childhood life stages. Though there is some variation at young ages due to uncertainty in the bioavailability and ingestion of Pb in soil and dust, the predictions trend towards convergence as the modeled child ages. Notably, although a dietary intake of 5 µg/day exceeds the current U.S. FDA interim reference value of 2.2 µg/day, the predicted BLLs from dietary intake are all well below the current CDC BLRV (Figure 2). The aggregated contribution of other sources of Pb (water, soil, and dust) concurrent with dietary intake are assessed probabilistically in the second phase of our study. 

### 3.3. IEUBK Probabilistic Exposure Pathway Analysis

**Figure 3:**
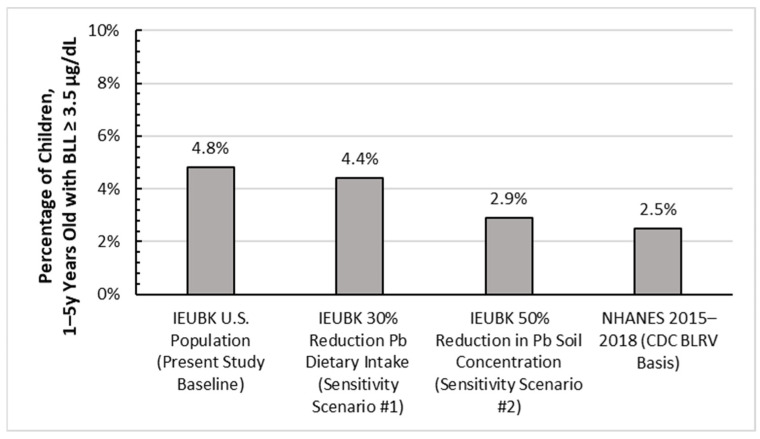
Comparison of percentage of children aged 1 to 5 years predicted to have BLLs ≥ 3.5 µg/dL as compared to the 2.5% exceedance fraction found in CDCs analysis of NHANES 2015–16 and 2017–18 data [43].

Probabilistic distributions of predicted BLLs using the IEUBK model were estimated for a baseline scenario as well as two reduction scenarios, where dietary Pb intake was reduced by 30% (in response to FDA proposed action level) or soil Pb concentration was reduced by 50% from the baseline (recent trends in soil concentration between AHHS and AHHS II) (Table 4 and Figure 3). The dietary reduction scenario reflects the decrease in dietary Pb intake expected to occur if the U.S. FDA’s action levels on Pb in baby food products were enforced. For children aged 1–2 years and 1–5 years, there was an approximately 8% reduction in the percent of children with a BLL exceeding 3.5 µg/dL (8.86% and 8.3%, respectively). Except for the 90th percentile, greater percentage reductions were observed among children aged 1–5 years, compared to children aged 1–2 years. Overall, the anticipated reductions in BLL from implementation of an action level were modest.

The second scenario evaluating a 50% reduction in soil Pb concentration was intended to represent the apparent decrease in U.S. residential soil Pb levels in homes with young children between 2005 and 2019. The most notable impacts on BLLs were in the percentage of children with predicted central tendency BLLs exceeding 3.5 or 5 µg/dL. For example, the predicted percentage of BLL’s greater than 3.5 µg/dL for children ages 1 to < 5 years was 4.8%, 4.4%, and 2.9% for the baseline, 30% food Pb intake reduction scenario, and 50% soil concentration reduction scenario, respectively. Thus, while food policy or reduction in lead intake from soil or dusts may have similar benefits for the average child, it is likely that measures such as residential Pb hazard abatements and lead-safe renovations will more noticeably benefit children with BLLs exceeding the current CDC BLRVs. 

**Table 4:** Distributions of predicted BLLs (µg/dL) using the IEUBK model for a national baseline scenario, a hypothetical policy reducing Pb in baby food intake by 30%, and a scenario evaluating a 50% reduction in soil Pb concentration, as compared to reported NHANES BLLs.

NHANES or Model BLL Distribution	Age	Monte Carlo Distributions					
		Distribution of IEUBK Predicted BLLs (µg/dL)					
		Mean (SD)	GM (GSD)	25th	50th	75th	90th
IEUBK National Baseline	1–2	1.8 (1.8)	1.4 (1.8)	0.95	1.3	1.9	3.1
IEUBK 30% Reduction Pb Diet Intake	1–2	1.6 (1.8)	1.3 (1.9)	0.82	1.2	1.7	2.8
IEUBK 50% Reduction in Pb Soil Conc.	1–2	1.6 (1.3)	1.3 (1.8)	0.88	1.2	1.8	2.6
NHANES 2011–2016 ^a^	1–2	--	0.9; CI: 0.9, 1.0	--	--	--	--
IEUBK National Baseline	1–5	1.6 (1.5)	1.3 (1.8)	0.85	1.2	1.7	2.6
IEUBK 30% Reduction Pb Diet Intake	1–5	1.4 (1.5)	1.1 (1.9)	0.72	1.0	1.5	2.4
IEUBK 50% Reduction in Pb Soil Conc.	1–5	1.4 (1.1)	1.2 (1.7)	0.80	1.1	1.6	2.3
NHANES 2009–2014 ^b^	1–6	1.3 (1.5)	1.0 (1.9)	--	0.85	--	--
NHANES 2015–2016 ^c^	1–5	--	0.76	--	0.69	1.1	1.9
NHANES 2017–2018 ^c^	1–5	--	0.67	--	0.62	1.1	1.9

^a^ As reported in Egan et al. (2021) [41]. ^b^ As reported in Zartarian et al. (2017) [22]. ^c^ CDC National Report on Human Exposure to Environmental Chemicals, 2011–2018 [42].

**Figure 4:**
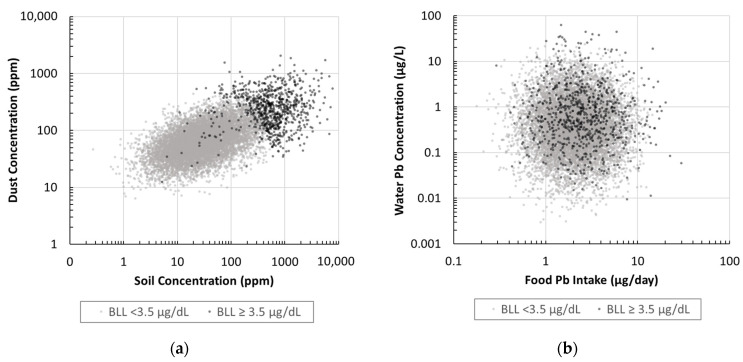
(**a**) Concurrent soil and dust concentrations associated with BLLs above or below 3.5 µg/dL for child 1–2 years old; (**b**) Concurrent water concentration and food intake associated with BLLs above or below 3.5 µg/dL for child 1–2 years old.

The relationship between source concentrations or intake rates and corresponding BLL estimates is visualized in Figure 4. Children with BLLs greater than 3.5 µg/dL tend to have had exposure to higher soil and dust concentrations. Furthermore, there is no clear trend between water concentrations or food intake with the higher BLLs. Therefore, soil and dust are important factors which often considerably influence the BLL whereas water and food sources are likely to have been a small contribution and have little influence over the BLLs of young children.

### 3.4. Sensitivity Analysis for IEUBK Exposure Pathways

**Figure 5:**
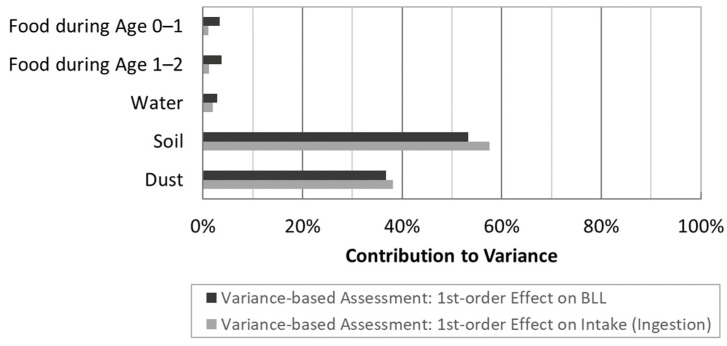
Contribution of sources to variance of BLL for children ages 1 to <2 normalized to total variance of 100%.

The contribution of environmental sources to variance in ingestion and BLL is demonstrated in Figure 5. The percentages were normalized to a total of 100%. The most notable outcome was that over 50% of the variance in both the ingestion and BLL was explained by uncertainty in the soil exposure and approximately 35% was explained by uncertainty in the dust exposure. The remaining sources (water, food during first year, and food during second year) explained less than 15% of the variance combined. 

## 4. Discussion

**Figure 6:**
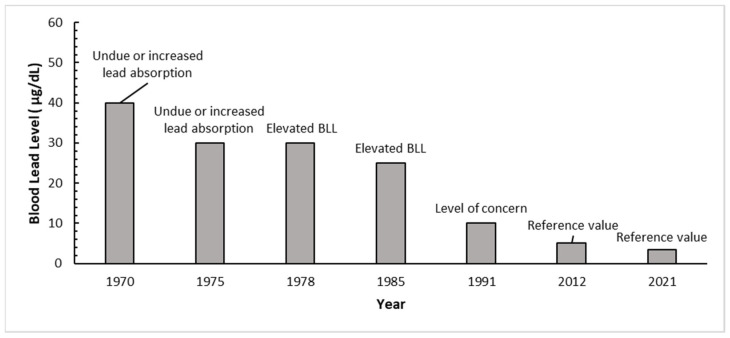
CDC BLL interpretations over time (adapted from Ruckart et al., 2021 [43]).

This paper presents an updated model comparison and evaluation of likely incremental changes in early childhood BLLs related to population changes in Pb environmental concentrations and/or dietary intake. A critical evaluation of relatively small (<0.5 µg/dL) incremental BLL changes is emerging as an important consideration for well-informed Pb risk management and policy because long-term biomonitoring of BLLs in the United States has shown a decrease in the geometric mean concentration in children aged 1–5 from 15 µg/dL in the 1976–1980 cycles to 0.83 µg/dL in the period of 2011–2016, or approximately a 94% decrease [41]. For example, the recent CDC BLRVs are intended to help clinical practitioners identify children with BLLs exceeding that of most children. The previous CDC BLRV of 5 µg/dL for children ages 1 to 5 referencing NHANES 2007–2008/2009–2010 data was lowered to 3.5 µg/dL in 2021 based on the 97.5th percentile of BLLs recorded in the NHANES 2015–2016/2017–2018 cycles [44]. These decreases have been reflected in dramatic decreases in CDC criteria for interpreting BLL results since 1970 (Figure 6). The consistent reductions in BLL reflect the effectiveness of federal measures to eliminate uses of Pb in gasoline, paint, plumbing, and other consumer products; however, it suggests that future reductions in population BLLs may be more modest due to the ubiquity of Pb in the environment.

Overall, we found that the current FDA dietary conversion factor may somewhat underestimate the unit increase or decrease in BLL predicted by current U.S. EPA regulatory models by a factor of 2 for a µg/day unit change in dietary Pb intake in young children. Thus, reconsideration of updated age-specific factors may be beneficial for the alignment of federal agency policy between the U.S. EPA mitigation of environmental contamination and U.S. FDA mitigation of adulterated food. Additionally, when considering the current U.S. population, it appears that variation in soil and dust Pb concentrations are particularly contributary to overall variance in observed BLLs. Thus, policy actions related to soil and dust remediation may continue to contribute more to reductions in upper bound BLLs observed in the population than policies addressing Pb intake from water or food. Conversely, as the number of remaining Pb-contaminated media in residences and commercial spaces occupied decreases, the relative importance of food and water as a determinant may increase in the future.

### 4.1. Dietary Conversion Factor 

The dietary conversion factor, or biokinetic slope factor, has been used to estimate BLLs in adults and children for both research and regulatory purposes [12,45,46]. Compartmental models approximate biokinetics by defining 10 or more compartments most often characterized by first-order transfer processes, with non-linear kinetics focused on a few key compartments. In contrast, slope factor models simplify Pb biokinetics by estimating the slope of an assumed linear relationship between either incremental Pb intake or uptake and the BLL. Such models are only reliable when intake levels are below saturation levels for gastrointestinal absorption and/or transfer to red blood cells, and thus are most often used to characterize non-occupationally exposed populations. An uptake slope factor is equivalent to the intake slope factor divided by the route-specific absorption fraction. Unlike the intake slope factor, the uptake slope factor is independent of administration route or substrate (e.g., water, soil, food). The previously mentioned FDA slope factor for Pb (0.16 µg/dL per ug/day) is based on dietary intake of Pb. If dietary Pb was assumed to have an absorption fraction of 0.5, the uptake slope factor equivalent to the FDA slope factor would be 0.32 µg/dL per ug/day (0.16/0.5). Interestingly, for typical dietary doses, U.S. EPA’s IEUBK Pb Model, although it is a compartmental model and thus more complex than a slope factor, generates an uptake slope factor of approximately 0.35 µg/dL per ug/day.

### 4.2. Lead Absorption Considerations

The absorption fraction of Pb in food is implicit in the intake slope factor employed by FDA to establish dietary guidelines. Many factors influence the absorption of dietary Pb. Among the most important is host age. It is well established that young children absorb Pb more efficiently than adults. What is understood to a lesser degree is the temporal element governing the transition of children as they grow into less efficient absorbers as they age. An intake slope factor implicitly assigns the same absorption fraction over the age range of its intended use. Biokinetic models such as the AALM and ICRP have decreasing absorption fractions over the lifetime of an individual. Specifically, the gastrointestinal absorption fraction for the ICRP model varies from 45% to 15%, and the AALM absorption fraction varies from 39% to 12%. While EPA’s IEUBK Model sets the absorption fraction for dietary Pb at 50% (0.5) over its entire 0-to-7-year age range, it is possible to adjust the absorption fraction. Such an absorption fraction adjustment is often implemented for soil-borne Pb based on in vitro bioaccessibility tests; however, it is uncommon to adjust the absorption fraction for dietary Pb. 

The food matrix can have an impact on Pb absorption. The infants in the Ryu et al. (1983) [11] absorbed substantial quantities of Pb from formula or whole milk in Pb-soldered cans. Lead introduced into the gastrointestinal tract in a solubilized form will be more bioaccessible and readily absorbed than Pb compounds in solid matrices [47]. The bioavailability of lead is reflected in the EPA IEUBK Pb Model default absorption fractions for water (50%—same as dietary) versus the absorption fraction for soil-borne Pb, which is 30%. On the other hand, the ICRP model includes only a general gastrointestinal absorption fraction (45% to 15%, decreasing with age), and does not differentiate between absorption fractions for dietary Pb and soil-borne Pb. The AALM includes a general gastrointestinal absorption fraction (39% to 12%, decreasing with age), as well as a relative bioavailability for soil and dust of 60%. 

Nutritional status is another factor that can influence the absorption of Pb in diet. Calcium, iron, and zinc are among the nutrients impacting Pb absorption. However, the effects of calcium are most prominent. Lead (like calcium, a divalent cation) “exploits” homeostatic mechanisms intended for calcium transport via the calcium-binding protein, calbindin [48]. Consequently, calcium deficiency promotes more opportunity for Pb absorption through this carrier-based uptake mechanism. As children age and move from liquid to solid diets, both factors serve to reduce the dietary absorption fraction of Pb. Consequently, for younger children, the U.S. FDA slope factor of 0.16 µg/dL per ug/day Pb intake may underestimate the response. 

### 4.3. Model Comparison and Evaluation

We found good agreement between models except for ICRP Version 4, which had not been calibrated for children. Our findings are consistent with historical and more recent model evaluation studies. Version 5 of the ICRP model and AALM have been shown to agree well with available blood Pb data [49,50]. More recently, the IEUBK Version 2 model was compared to data from a population of children near the Bunker Hill Superfund Site which included paired estimates of BLL and Pb concentration in environmental media. The model explained 90% of the variance in BLLs and the modeled geometric mean was within 0.3 µg/dL of the empirically observed age stratified geometric means [51]. 

### 4.4. IEUBK Probabilistic Assessment

Overall, the reasonable agreement between our probabilistic BLL predictions and recent NHANES biomonitoring assessments suggests that separating MonteCarlo analysis of environmental Pb concentrations and dietary Pb input from inter-human biokinetic variability is useful for risk management. Notably, IEUBK slightly overpredicted BLLs at each percentile, which was not surprising because our probabilistic assessment relied on soil and dust concentrations reported in the 2005–2006 AHHS. The recently completed AHHS II (2018–2019) suggests soil Pb concentrations have decreased on average by 50% in homes with children under the age of 6. We chose to use the earlier AHHS soil and dust data published in Zartarian et al. (2017) [22] for our study because Pb accumulation in dust in AHHS II was reported as µg/ft^2^ loading, rather than ppm concentration reported in Zartarian et al. (2017) [22] as required by the models. Overall, in homes with and without young children, the average soil concentration appears to have decreased from 169 ppm (95% CI: 132 to 237 ppm) in AHHS to 106 ppm (95% CI: 77 to 134 ppm) for all soils. Mean floor dust loadings were similar (3.7 versus 3.6 µg/ft^2^), but risk management interventions appear to have decreased window sill loadings from 156 to 54 µg/ft^2^ in AHHS and AHHS II, respectively. In principle, at current dietary intakes of Pb less than 5 µg/day, characterizing variation in environmental media and food intake appears to be sufficient to explain most of the variation in population BLLs, assuming that intake and biokinetic variation are independent, as was recently assumed by Zartarian et al. (2017) [22].

### 4.5. Source Variance Contributions

In the present study, the majority of the variance observed in infant BLLs was attributed to soil and dust exposure, while Pb from water and food during the first and second years of life explained only 15% of the variance in infant BLLs. This finding is congruent with other research utilizing exposure data from the U.S. Zartarian et al. (2017) [22], which determined that soil and dust were the primary source of exposure for infants. The authors found that, when aggregate exposures were considered, the 97.5th percentile of their modeled distribution exceeded the CDC BLRV of 3.5 µg/dL even when the water lead level was assumed to be 0 ppb Pb. The observed appreciable contribution of soil and dust to BLL at the upper bound aligns with our findings that water lead level is a minor contributor to BLLs in children when thresholds are exceeded (with the exception of unique site-specific conditions leading to unusually high exposure to lead in water). Additionally, food was determined to be a background source of exposure in children 0 to 6 months of age, but a major source of exposure for children 1 to 2 years of age [22]. In the present study, however, food was estimated to have a much smaller contribution to infant BLL and was a background contributor to variance for both children aged 0 to 1 years and 1 to 2 years. The importance of soil and dust as a primary contributor to BLLs in young children is also reflected in a previous analysis by Goodrum et al. (1996) [22], in which soil and dust explained 33% and 45%, respectively, of the BLL variance in children aged 2 to 3 years when Pb-based paint and water Pb exposures are negligible. 

The importance of setting related to source contributions of BLLs is apparent when considering global trends. In France, a study predicting infant and child BLLs using the O’Flaherty PBTK model found that food, specifically vegetable and milk products, were the primary contributors to total Pb ingestion for children under 2 years [52]. Soil, on the other hand, was estimated to be a background source of exposure for children under 2 in France [52]. However, the authors noted that soil contamination data was lacking in some geographic areas, including around known legacy sites of contamination [52], which may have increased the uncertainty regarding the contribution of soil Pb on child BLLs. In Germany, Hahn et al. (2022) [53] measured variance contributors through blood Pb measurements of children aged 3 to 17 years, tap water sampling, and food-frequency surveys. Lead intake via domestic tap water was determined to be the strongest contributor to participants’ BLLs [53]. While soil and dust contributions were not measured, the authors noted that 69% of the variance in participants’ BLLs was unexplained by the model [53]. In Mexico City, an analysis of five birth cohorts of children aged 1 to 5 years showed that elevated child BLLs were associated with Pb in air and the use of Pb-glazed ceramics [54]. The authors noted that the use of Pb glazes and Pb-glazed ceramics is common in Mexico [54]. This further highlights the influence of setting and cultural factors on child Pb exposure. 

### 4.6. Strengths and Limitations

The main strengths of this study are the use of state-of-the-art IEUBK and AALM biokinetic models to evaluate contributors to incremental increases or decreases in childhood BLLs. For example, IEUBK has been carefully verified and evaluated historically (e.g., Zaragozal and Hogan et al., 1998 [55]) and in the most recent update to Version 2.0 (e.g., Brown et al., 2023 [51]). Another strength of our analysis is the visualization of the differences in determinants of exposure for BLLs greater than 3.5 µg/dL, which appear to be disproportionately affected by soil and dust concentrations. Nonetheless, limitations apply to the use of biokinetic models in this and prior studies. For example, our study focused on variation in environmental media concentrations as key determinants of variation in population BLLs, whereas other studies such as the probabilistic assessment of Zartarian et al. (2017) [22] considered independent contributions of both biokinetic (with measurement uncertainty) and exposure variances. We focused on variance in exposure because risk mitigation measures are often available for exposure pathway modification, whereas inter-human variation in biokinetic response cannot typically be modified, except for nutritional status. Most of the material limitations in the models involve knowledge gaps in the estimation of target tissue concentrations in organs such as the kidney and brain, rather than in the prediction of blood Pb concentration [17,50]. One limitation specific to blood is that most of the models have been calibrated or evaluated using data reflecting relatively high Pb exposure levels [49,50,51]. However, we found good agreement between recently measured distributions of BLLs in the United States and the IEUBK model when correlated distributions of soil, dust, and water concentration, as well as food intake, were considered. 

### 4.7. Future Research

Blood Pb concentrations have been consistently decreasing for over the last five decades (Figure 6), which has increased the importance of reliable measures of Pb in environmental media, including food. A recent systematic review and meta-analysis of concentrations found the availability of high quality and consistent data is relatively limited except for air, which typically contributes very little towards observed BLLs in the U.S. [56]. Thus, it has been suggested by the authors of the review that the centralized reporting including standardized sample collection, analysis, and reporting for soil, water, and food be developed using the principles that have been applied in the development and implementation of the U.S. EPA Air Quality Monitoring System (AQS) database. The need for standardized data collection is further evidenced by Parker et al. (2022) [57], who noted a variability in Pb concentrations within singular baby food products and suggested that risk estimates based on maximum measured exposure concentrations likely overestimate risk associated with baby food products. Future analyses of Pb in food should use procedures that can achieve low limits of detection [3]. Our study found that based on available data, tap water is unlikely to explain exceedance of BLRVs in the context of current food Pb intake, as well as dust and soil concentrations. In contrast, it was suggested approximately one decade ago by Triantafyllidou et al. (2014) [58] that lead intake from water could be contributory towards exceedance of blood lead thresholds. As noted by Stanek et al. (2020) [59], formula-fed infants might receive as much as 80% of their Pb intake from water. Because infants younger than 6 months are recommended to consume primarily breast milk and/or formula, and do not participate in activity patterns such as crawling and hand-to-mouth activities, it is important to consider water as a significant source of Pb exposure in infants 6 months and younger. International studies in Germany, Australia, and Canada have suggested that Pb levels in cow’s milk may be higher than in human milk, and thus infant formula should not be ruled out as a potential source of lead to infants [60]. The ongoing decrease in population BLLs (Figure 6) and potential uncertainty in relative source contributions further emphasizes the importance of a robust and centralized framework for collection and reporting of current environmental concentrations of lead. Although water and food may become proportionately more important in the future as soil and dust hazards are more completely mitigated, our probabilistic analysis suggests that remediation of structural lead hazards in residential and commercial spaces occupied by children should remain a near-term priority. Additionally, as BLRVs continue to decrease, there may be a benefit to increased development of age-specific dietary conversion factors for lead, which could be incorporated in future U.S. FDA reference values for foods consumed by children.

## Data Availability

The data presented in this study are available on request from the corresponding author.
